# A Pheochromocytoma With Adrenocorticotropic Hormone Secretion and Subsequent Fatal Outcome: A Case Report

**DOI:** 10.7759/cureus.77459

**Published:** 2025-01-15

**Authors:** Hiroko Nakabayashi, Masaru Akiyama, Takuma Yodokawa, Wataru Itoh, Akihiko Taguchi, Komei Takeda, Naho Yamamoto, Takushi Morishige, Eiji Ikeda, Yasuharu Ohta

**Affiliations:** 1 Department of Endocrinology, Metabolism, Hematological Science and Therapeutics, Yamaguchi University Graduate School of Medicine, Ube, JPN; 2 Department of Diabetes and Endocrinology, Yamaguchi Prefectural Grand Medical Center, Hofu, JPN; 3 Department of Pathology, Yamaguchi University Graduate School of Medicine, Ube, JPN

**Keywords:** acth, cushing’s syndrome, ectopic acth-secreting tumor, osilodrostat, pheochromocytoma

## Abstract

Hypercortisolemia and hypercatecholaminemia in ectopic adrenocorticotropic hormone (ACTH)-secreting pheochromocytoma represent life-threatening conditions, particularly when diagnosis is delayed. In this case, a 66-year-old male patient with poorly controlled diabetes presented with severe constipation in the absence of the classic Cushingoid phenotype. Computed tomography revealed bilateral adrenal masses, and ^123^I-metaiodobenzylguanidine scintigraphy revealed radiotracer uptake in the left adrenal gland. Endocrine assessments confirmed elevated catecholamine, ACTH, and cortisol levels, indicating an ACTH-secreting pheochromocytoma. Despite preoperative management with osilodrostat and alpha-blockers, the patient experienced sudden cardiopulmonary arrest and succumbed. This case highlights that prompt diagnosis and intervention are particularly important in ACTH-secreting pheochromocytoma.

## Introduction

Pheochromocytomas are rare catecholamine-secreting tumors arising from chromaffin tissue, with an overall incidence of 0.4-2.1 cases per million people [1]. These tumors are occasionally associated with ectopic production of various hormones or cytokines [2]. Cushing’s syndrome, a condition characterized by cortisol hypersecretion, is rarely caused by ectopic adrenocorticotropic hormone (ACTH)-secreting neuroendocrine tumors. The primary sources of ectopic ACTH production include bronchial carcinoid tumors, lung cancers, and medullary thyroid cancers [3]. Pheochromocytoma accounts for 2%-6% of neoplastic cases of ectopic Cushing’s syndrome [4]; approximately 1% of pheochromocytoma cases are associated with ectopic ACTH production [5]. However, the general awareness of ACTH-secreting pheochromocytoma seems relatively low among general physicians.

Surgery is the primary treatment for most patients with Cushing’s syndrome; however, when surgery is contraindicated or unsuccessful, or while waiting for surgery, steroidogenic inhibitors are sometimes used to control hypercortisolemia to improve postoperative recovery and wound healing and reduce the risk of infection [6]. Metyrapone prevents cortisol synthesis by inhibiting 11 β-hydroxylase, the enzyme responsible for the conversion of deoxycortisol to cortisol, and is frequently the first choice of medication due to its rapid onset. Osilodrostat has recently become an available treatment option for Cushing’s syndrome in Japan. Like metyrapone, it is an 11-β-hydroxylase inhibitor, but it is reported to have a longer duration of action. In the case presented here, osilodrostat was administered through a gastric tube to a patient with ACTH-secreting pheochromocytoma. Although the patient eventually died, a certain degree of osilodrostat treatment efficacy was observed.

Given the significant morbidity associated with both Cushing’s syndrome and pheochromocytomas [7,8], delayed diagnosis and treatment can lead to catastrophic outcomes, and prompt diagnosis and early intervention tailored to the patient's condition are of the utmost importance. Herein, we report a patient with an ACTH-secreting pheochromocytoma who ultimately succumbed to the disease, partly due to the delayed diagnosis. This case underscores the importance of increasing awareness of this rare condition.

## Case presentation

**Figure 1:**
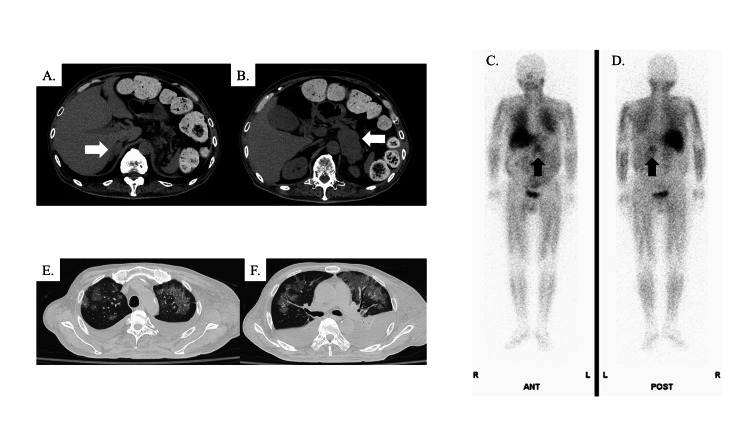
Image findings. (A,B) Abdominal plain CT finding. Arrows show right (A) and left (B) adrenal masses. 123I-metaiodobenzylguanidine scintigraphy finding from anterior side (C) and posterior side (D). Arrows show accumulation in the left adrenal gland. (E,F) Chest CT (lung window setting) CT: computed tomography

A 66-year-old male patient with a history of hypertension, type 2 diabetes mellitus, dyslipidemia, and hyperthyroidism presented with poor glycemic control (hemoglobin A1c: 8.8%) and severe constipation lasting for one month. He was urgently admitted to the hospital with suspected ileus. Computed tomography (CT) revealed significant stool accumulation but no ileus, and bilateral adrenal masses were detected (Figures 1A, 1B); elevated levels of urinary and plasma catecholamines, metanephrine, and normetanephrine were also detected, leading to a diagnosis of pheochromocytoma. Severe constipation was considered to be caused by excessive catecholamine secretion by the pheochromocytoma. In addition, endocrinological analysis showed elevated ACTH and cortisol levels, despite magnetic resonance imaging ruling out a pituitary tumor; this finding was initially considered to be the result of stress responses. ^123^I-metaiodobenzylguanidine (MIBG) scintigraphy revealed accumulation only in the left adrenal gland (Figures 1C, 1D).

Despite aggressive treatment with α-blockers, the patient's condition deteriorated, manifesting as massive systemic edema and refractory cholecystitis. Treatment with intravenous immunoglobulin for hypogammaglobulinemia concurrent with severe infection led to rapid respiratory failure requiring mechanical ventilation. CT revealed ground-glass opacities and nodular infiltrates in both lungs, along with pleural effusion (Figures 1E, 1F). β-D-glucan was elevated (208.5 pg/mL, reference range < 11.0 pg/mL), and a peripheral blood cytomegalovirus (CMV) antigen immunological assay using the horseradish peroxidase-labeled monoclonal antibody was marginally positive (eight CMV-antigen positive leukocytes per 50,000 cells). In response, steroid pulse therapy, trimethoprim-sulfamethoxazole, and ganciclovir were initiated suspecting the possibility of blood product transfusion-related acute lung injury, pneumocystis pneumonia, or CMV pneumonia. The patient’s lung lesions progressively improved. Given the urgent need for intervention for the pheochromocytoma, the patient was weaned off mechanical ventilation and transferred to our hospital, which is designated as a hospital with specific functions.

**Figure 2:**
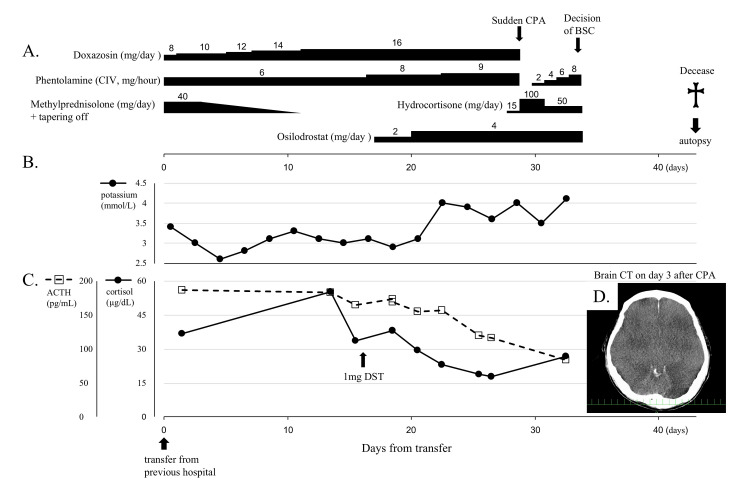
Patient’s clinical course from the time of transfer to our hospital. (A) The time courses of treatment by α-blockers and osilodrostat for pheochromocytoma and management of steroid hormone. Line graphs illustrating changes in potassium (B), cortisol, and ACTH (C) levels. (D) Brain CT on day 3 after CPA ACTH: adrenocorticotropic hormone; CPA: cardiopulmonary arrest; BSC: best supportive care; DST: dexamethasone suppression test; CT: computed tomography; CIV: continuous intravenous infusion

The patient’s clinical course is shown in Figure 2. Upon admission, the treatment regimen included 8 mg/day oral doxazosin and continuous intravenous phentolamine to manage catecholamine hypersecretion, along with 40 mg/day intravenous methylprednisolone for lung injury. The trimethoprim-sulfamethoxazole therapy was maintained at a prophylactic dose, and ganciclovir was discontinued at a previous hospital. The β-D-glucan concentration decreased to 59.3 pg/mL (reference range <11.0 pg/mL), and the CMV antigen was negative. The patient was not allowed oral intake and was managed with high-calorie intravenous nutrition and oral medication delivered using a gastric tube. Physical examination revealed a height of 166 cm, weight of 89.8 kg (a 15 kg increase in recent months), a body temperature of 36.2 °C, blood pressure of 112/68 mmHg, heart rate of 98 bpm, and oxygen saturation of 98% with 5 L/minute of supplemental oxygen administered by mask. Notable findings included generalized skin hyperpigmentation, a markedly distended and firm abdomen, and widespread edema. The patient was bedridden and exhibited significant muscle weakness.

Laboratory tests revealed an elevated white blood cell count with predominant neutrophilia, marked lymphopenia, anemia, thrombocytopenia (Table 1), and reduced immunoglobulin levels that correlated with lymphocyte depletion. Mild hypokalemia was also observed. C-reactive protein was not significantly increased (Table 2). Cholecystitis had been apparent at a previous hospital; however, laboratory findings upon transfer to our facility did not show any indicators of an active infection. The endocrine evaluation revealed significantly elevated levels of catecholamines, ACTH, and cortisol despite methylprednisolone administration (Tables 3, 4).

**Table 1:** Complete blood count Neutro: neutrophils; Eosino: eosinophils; Baso: basophils; Lymph: lymphocytes; Mono: monocytes; RBC: red blood cells; Ht: hematocrit; MCV: mean corpuscular volume; MCH: mean corpuscular hemoglobin; MCHC: mean corpuscular hemoglobin concentration; Plt: platelets

Complete blood count	Result	Reference range
WBC (×10^6^/L)	13,940	3,300-8,600
Neutro (%)	97.7	38.5-80.5
Eosino (%)	0	0-8.5
Baso (%)	0.1	0-2.5
Lymph (%)	0.8	16.5-49.5
Mono (%)	1.4	2.0-10.0
RBC (×10^10^/L)	246	435-555
Hb (g/dL)	7.5	13.7-16.8
Ht (%)	22.4	40.7-50.1
MCV (fL)	91.1	83.6-98.2
MCH (pg)	30.5	27.5-33.2
MCHC (%)	33.5	31.7-35.3
Plt (×10^10^/L)	3.6	15.8-34.8

**Table 2:** Biochemistry CRP: C-reactive protein; TP: total protein; Alb: albumin; T-Bil: total bilirubin; AST: aspartate aminotransferase; ALT: alanine aminotransferase; LDH: lactate dehydrogenase; ALP: alkaline phosphatase; γ-GTP: γ-glutamyl transpeptidase; CPK: creatinine kinase; BUN: blood urea nitrogen; Cr: creatinine; eGFR: estimated glomerular filtration rate; UA: uric acid; Na: sodium; K: potassium; Cl: chloride; Ca: calcium; P: phosphorus; Glu: glucose; IgG: immunoglobulin G; IgA: immunoglobulin A; IgM: immunoglobulin M; ChE: cholinesterase

Biochemistry	Result	Reference range
CRP (mg/dL)	0.27	0-0.14
TP (g/dL)	4.1	6.6-8.1
Alb (g/dL)	2.5	4.1-5.1
T-Bil (mg/dL)	1.2	0.4-1.5
AST (U/L)	386	13-30
ALT (U/L)	934	10-42
LDH (U/L)	687	124-222
ALP (U/L)	525	38-113
γ-GTP (IU/L)	430	13-64
ChE (U/L)	97	240-486
CPK (U/L)	831	29-248
BUN (mg/dL)	64	8-20
Cr (mg/dL)	1.11	0.65-1.07
eGFR (mL/minute/1.73 m^2^)	52	-
UA (mg/dL)	5.6	3.7-7.8
Na (mmol/L)	151	138-145
K (mmol/L)	3.4	3.6-4.8
Cl (mmol/L)	113	101-108
Ca (mg/dL)	7.3	8.8-10.1
P (mg/dL)	3.6	2.7-4.6
Glu (mg/dL)	137	73-109
IgG (mg/dL)	231	861-1,747
IgA (mg/dL)	41.7	93-393
IgM (mg/dL)	27.6	33-183

**Table 3:** Endocrinological examination ACTH: adrenocorticotropic hormone ^*^Under administration of 40 mg/day of methylprednisolone

Endocrinological examination	Result	Reference range
Free metanephrine (pg/mL)	287	0-130
Free normetanephrine (pg/mL)	921	0-506
Adrenalin (pg/mL)	2,210	0-100
Noradrenalin (pg/mL)	6,218	100-450
Dopamine (pg/mL)	926	0-20
ACTH (pg/mL)^*^	186.3	7.2-63.3
Cortisol (µg/dL)^*^	36.7	4.4-21.1

**Table 4:** Urine test ^*^Under administration of 40 mg/day of methylpredonisolone

Urine test	Result	Reference range
Free metanephrine (mg/day)	2.21	0.04-0.19
Free normetanephrine (mg/day)	1.65	0.09-0.33
Adrenalin (µg/day)	915	3.4-26.9
Noradrenalin (µg/day)	1,764.4	48.6-168.4
Dopamine (µg/day)	678.1	365-961.5
Cortisol (µg/day)^*^	634.9	4.3-176

A diagnosis of pheochromocytoma was confirmed based on catecholamine hypersecretion (Tables 3, 4) and the presence of a ^123^I-MIBG scintigraphy-positive tumor. Persistent elevation of ACTH and cortisol despite steroid therapy (40 mg of methylprednisolone) raised suspicion of ACTH-secreting pheochromocytoma. An overnight 1-mg dexamethasone suppression test was performed after discontinuation of steroid treatment, after which cortisol levels remained high (Table 5), leading to a diagnosis of ACTH-secreting pheochromocytoma. Neutrophilic leukocytosis and mild hypokalemia supported this diagnosis, although there were no clinical features of Cushing's syndrome, and somatostatin receptor scintigraphy (^111^In-pentetreotide) was negative for adrenal masses.

**Table 5:** Result of the overnight 1-mg dexamethasone suppression test ACTH: adrenocorticotropic hormone

Dexamethasone suppression test	Before	1 mg
ACTH (pg/mL)	164.3	173.1
Cortisol (µg/dL)	33.5	38

Consultation with the urology and anesthesiology departments concluded that treatment for hypercortisolism, correction of nutritional deficiencies and mineral abnormalities, and infection management were necessary before surgical intervention. Treatment included gradual escalation of doxazosin and phentolamine, tapering of methylprednisolone, and initiation of osilodrostat through a gastric tube on day 17 to control hypercortisolemia (Figure 2A). Osilodrostat therapy suppressed the secretion of cortisol to some extent. Interestingly, it lowered not only cortisol but also ACTH levels and successfully corrected potassium levels (Figures 2B, 2C), with the subsequent initiation of hydrocortisone replacement to avoid hypoadrenalism. The β-D-glucan level decreased to 5.8 pg/mL within a normal range (reference range <11.0 pg/mL), and the CMV antigen remained negative.

Unfortunately, on day 29 after the transfer, the patient experienced sudden cardiopulmonary arrest of an unknown etiology. Despite successful resuscitation after more than 10 minutes of arrest, brain CT on day 3 postarrest revealed severe cerebral edema (Figure 2D), indicating a poor prognosis for neurological recovery. The best supportive care was provided; however, the patient died 42 days after the transfer.

**Figure 3:**
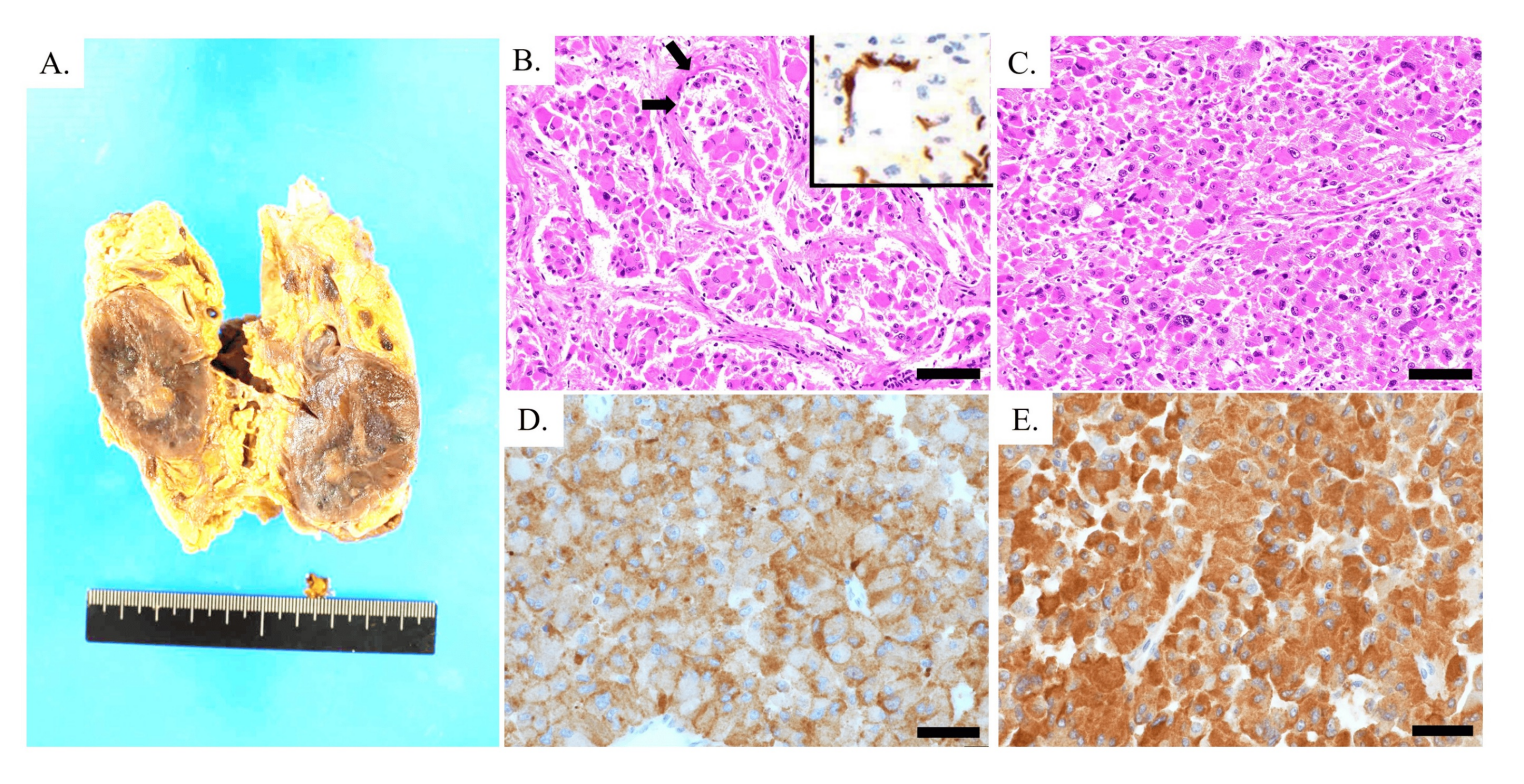
(A) Macroscopic image of the left adrenal tumor. A circumscribed mass was found in the left adrenal gland. (B-E) Microscopic images of left adrenal tumor. Atypical cells showing polymorphism grow in alveolar (B) and diffuse (C) patterns with sustentacular cells (arrows) (B), which are highlighted by immunohistochemical staining for S-100 (inset in B). Tumor cells are immunopositive for synaptophysin (D) and chromogranin A (E). Hematoxylin-eosin stain (B,C); immunostains for synaptophysin (D) and chromogranin A (E). Scale bars: 100 μm (B,C) and 40 μm (D,E)

A subsequent autopsy confirmed a 4 cm × 2.5 cm tumor in the left adrenal gland (Figure 3A). Histologically, the tumor comprised atypical cells with pleomorphic nuclei, enlarged round nuclei, and multinucleated giant cells that proliferated in alveolar (Zellballen) (Figure 3B) and diffuse (Figure 3C) patterns. At the periphery of the alveolar nests, S-100-positive sustentacular cells were observed (arrows and inset in Figure 3B). Immunohistochemically, the atypical cells were positive for synaptophysin and chromogranin A (Figures 3D, 3E). Based on these findings, the tumor in the left adrenal gland was diagnosed as a pheochromocytoma.

**Figure 4:**
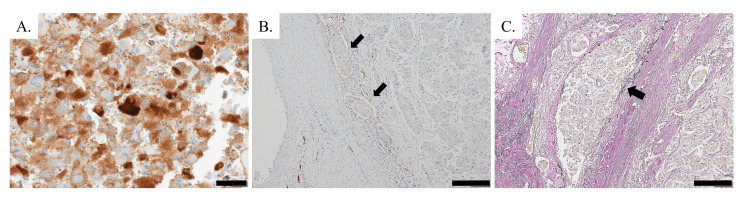
Production of ACTH and the invasive properties of left adrenal pheochromocytoma. (A) Immunohistochemical staining showing the production of ACTH. Invasive properties of tumor cells in lymphatic vessels (arrows) (B) and veins (arrow) (C). Immunostains for ACTH (A) and D2-40 (B); Elastica van Gieson stain (C). Scale bar: 40 μm (A) and 200 μm (B,C) ACTH: adrenocorticotropic hormone

Tumor cells were immunopositive for ACTH (Figure 4A), indicating that the tumor was an ACTH-secreting pheochromocytoma. Immunostaining for D2-40 and Elastica van Gieson revealed the invasive properties of the tumor cells in the lymphatic system and veins, respectively (Figures 4B, 4C). The tumor of the present case was classified as moderately differentiated with an intermediate risk according to the Grading System of Adrenal Pheochromocytoma and Paraganglioma (GAPP) [9]. In the right adrenal gland, there was a nodular lesion in which cells with rounded nuclei and clear-to-eosinophilic cytoplasm proliferated in an alveolar pattern, and the boundary between the nodule and the surrounding adrenal tissue was somewhat unclear. Immunohistochemically, the cells in the nodule on the right adrenal gland tested negative for ACTH, indicating it was not the source of ACTH and that the cortical hyperplasia was due to ACTH secretion from the left adrenal pheochromocytoma.

Although the autopsy revealed findings suggestive of catecholamine-induced cardiomyopathy, the exact cause of the patient's sudden cardiopulmonary arrest and subsequent death remains unclear. Peritonitis and mild myocardial hypertrophy were also noted but were not considered to be direct causes of death.

## Discussion

Here, we present a rare case of an ACTH-secreting pheochromocytoma that ultimately resulted in death. The outcomes of patients with ACTH-secreting pheochromocytomas are generally favorable, although they are often difficult to diagnose. A systematic review revealed that 88% of patients achieved long-term survival [10], with survival rates similar to those in other pheochromocytoma cohorts [11,12]. Pathological findings revealed lymphatic and venous invasion of the tumor with an intermediate-risk classification based on the GAPP scoring system. The five-year survival rate for intermediate-risk pheochromocytomas is 66.8% compared with 22.4% for high-risk tumors [9]. Among the patients who died, infection or infection-related complications were the most common causes of death [10], suggesting that hypercortisolemia likely plays a role in the risk of infection [13,14].

Cushing’s syndrome causes immunosuppression, increasing infection risk. Patients who die from Cushing’s syndrome are typically older, men, and more likely to have muscle weakness, diabetes mellitus, and ectopic Cushing’s syndrome than survivors [14]; many of these risk factors were observed in the present case. Cholecystitis had already been recognized, and pneumocystis pneumonia was suspected at a previous hospital. Autopsy findings confirmed peritonitis secondary to cholecystitis. Although peritonitis did not appear to be the direct cause of death, infections such as peritonitis and cholecystitis are likely to have contributed to the deterioration of the patient’s condition.

Surgical intervention is the primary treatment for hypercortisolism in Cushing’s syndrome, regardless of its etiology. However, medication is necessary if surgery is delayed, contraindicated, or unsuccessful. Cortisol-lowering therapy is sometimes used preoperatively to improve postoperative recovery and wound healing and reduce the risk of infection [6]. In the present case, it was expected that pharmacotherapy with a steroidogenic inhibitor would be necessary before surgery to reduce cortisol levels, correct electrolyte imbalances, and control infections. However, the patient was unable to take any oral medications. In Japan, metyrapone is commonly used as a steroidogenic inhibitor, but its administration using a gastric tube is generally considered inappropriate [15]. Osilodrostat, a tablet formulation, was approved for use in Japan in 2021 and is currently available for the treatment of Cushing's syndrome. As osilodrostat can be crushed, making its administration through a gastric tube easier than metyrapone, it was one of the reasons to select osilodrostat in this case. Osilodrostat administered using the gastric tube successfully reduced cortisol and normalized potassium levels (Figure 2B). Osilodrostat therapy also reduced both the cortisol and ACTH levels (Figure 2C). Previous reports suggested that metyrapone suppresses both cortisol and ACTH secretion in ACTH-secreting pheochromocytomas [16,17]. Such cases suggest a glucocorticoid-driven positive feedback loop between cortisol and ACTH secretion, leading to the hypersecretion of both hormones. The present case also supports the possibility of such feedback.

## Conclusions

We report a case of ectopic ACTH-secreting pheochromocytoma with a fatal outcome, though administration of osilodrostat using a gastric tube effectively suppressed both ACTH and cortisol levels. Although ACTH-secreting pheochromocytomas are rare, early diagnosis and treatment can lead to favorable outcomes. The present case suggests that early pharmacological intervention with adrenal steroidogenesis inhibitors, along with alpha-blockers, is useful for preoperative management. Raising awareness of this condition is critical to prevent delays in diagnosis and treatment.
